# Translation and validation of the multiple sclerosis walking scale 12 for the German population – the MSWS-12/D

**DOI:** 10.1186/s12955-023-02190-2

**Published:** 2023-10-09

**Authors:** Anna Chorschew, Firat Kesgin, Judith Bellmann-Strobl, Peter Flachenecker, Insa Schiffmann, Friederike Rosenthal, Patrick Althoff, Daniel Drebinger, Radina Arsenova, Ludwig Rasche, Eva-Maria Dorsch, Christoph Heesen, Friedemann Paul, Jan-Patrick Stellmann, Tanja Schmitz-Hübsch

**Affiliations:** 1 Experimental and Clinical Research Center (ECRC), a cooperation between Max-Delbrück-Center for Molecular Medicine in the Helmholtz Association and Charité - Universitätsmedizin Berlin, Lindenberger Weg 80, 13125 Berlin, Germany; 2 Experimental and Clinical Research Center, Charité - Universitätsmedizin Berlin, Universität Berlin and Humboldt Universität zu Berlin, Berlin, Germany; 3 Max-Delbrück-Center for Molecular Medicine in the Helmholtz Association (MDC), Berlin, Germany; 4 Institute of Neuroimmunology and Multiple Sclerosis (INIMS), Center for Molecular Neurobiology, University Medical Center Hamburg-Eppendorf, Hamburg, Germany; 5 Neuroscience Clinical Research Center, Charité - Universitätsmedizin Berlin, Universität Berlin and Humboldt Universität zu Berlin, Berlin, Germany; 6 Neurological Rehabilitation Center Quellenhof, Bad Wildbad, Germany; 7 Department of Neurology, University Medical Centre Hamburg-Eppendorf (UKE), Hamburg, Germany; 8 Department of Physical Medicine and Rehabilitation, Charité - Universitätsmedizin Berlin, corporate member of Freie Universität Berlin and Humboldt-Universität zu Berlin, Berlin, Germany; 9 Department of Pediatrics, St Joseph Krankenhaus Berlin-Tempelhof, Berlin, Germany; 10 Department of Psychiatry, Schlosspark-Klinik Charlottenburg, Berlin, Germany; 11 Department of Neurology, Charité - Universitätsmedizin Berlin, Freie Universität Berlin and Humboldt Universität zu Berlin, Berlin, Germany; 12 Aix-Marseille Univ, CNRS, CRMBM, UMR 7339; APHM La Timone, CEMEREM, Marseille, France; 13 APHM, Hospital de la Timone, CEMEREM, Marseille, France

**Keywords:** Multiple sclerosis, Walking ability, Gait impairment, MSWS-12, Patient reported outcome

## Abstract

**Background:**

Gait impairment is a relevant problem in persons with multiple sclerosis (pwMS). The Multiple Sclerosis Walking Scale 12 (MSWS-12) is a valid Patient Reported Outcome Measure (PROM) to evaluate walking ability in pwMS. The aim of this study was to provide a linguistically valid translation of MSWS-12 into German language (MSWS-12/D) and to evaluate its psychometric properties.

**Methods:**

The MSWS-12 was translated in a process modified from guidelines for the cross-cultural adaption of PROMs, and a pre-test was applied in a small sample of 20 pwMS to evaluate comprehensibility and acceptance. Psychometric properties (floor and ceiling effects, internal consistency, construct validity) were then assessed in 124 pwMS seen at academic MS centers. Construct validity was evaluated against Expanded Disability Status Scale (EDSS) and maximum gait speed in the Timed 25-Foot Walk (T25FW).

**Results:**

Although the sample covered a wide spectrum of symptom severity, the majority had rather low levels of disability (EDSS median 2.0) and 6.5% scored EDSS of 0. In this sample, MSWS-12/D showed floor effects (36% with score 0) and for internal consistency, a Cronbach’s alpha of 0.98 was calculated. MSWS-12/D score showed a relevant correlation to EDSS (ρ = 0.73) and T25FW speed (r=-0.72).

**Conclusion:**

We provide MSWS-12/D as a linguistically valid German version of MSWS-12. Psychometric properties (acceptance, floor and ceiling effects, internal consistency and construct validity) in pwMS were similar to those described for the original version. This indicates that MSWS-12/D can be applied as equivalent to the original version in German speaking pwMS. Results support the relevance of PROMs to capture patient perception of walking ability in addition to performance-based assessments such as maximum walking speed or maximum walking distance.

## Background

Multiple Sclerosis (MS) is an inflammatory and neurodegenerative disease of the central nervous system characterized by heterogeneous focal neurological symptoms. Motor deficits are common, with the majority of persons with MS (pwMS) developing gait impairment over the course of the disease [1–3]. The ability to walk safely and associated everyday mobility define a central aspect of quality of life among pwMS [1]. Therefore, assessment of gait capability in these persons is a relevant aspect of patient monitoring in clinical practice as well as in research contexts.

Accordingly, walking is the most important factor when rating pwMS with advanced levels of disability in the Expanded Disability Status Scale (EDSS) [4], the most widely used clinical rating scale for impairment in MS. Quantitative tests of ambulation include the Timed 25 Foot Walk (T25FW) for maximal walking capacity or 6 min Walk (6 MW) for gait endurance, which are commonly applied complementary to clinical ratings [5, 6]. However, those assessments require clinical resources, and it has been shown that both gait parameters are rather uncertain predictors of everyday mobility [7]. A general limitation of clinical tests is their inability to represent the patient’s perspective. For this reason, Patient Reported Outcome Measures (PROMs) are highly recommended in addition to clinical testing [8]. PROMs – such as questionnaires or scales - document the patients’ self-evaluation regarding specific aspects of their disease. The U.S. Food and Drug Administration (FDA) and the European Medicines Agency (EMA) demand the use of PROMS in pivotal trials for the approval of novel therapies [9, 10] to support results of clinical outcome assessments. Common measures of disease progression in pwMS such as Disease Steps (DS) [11] have been transformed into patient-reported forms like Patient Determined Disease Steps (PDDS), a 9-step rating scale considering patients’ perception of their disability level [12, 13].

In 2003, Hobart and colleagues developed the Multiple Sclerosis Walking Scale 12 (MSWS-12) as a PROM for gait ability in pwMS [2]. In this questionnaire patients are asked to rate their MS-caused impairment regarding twelve aspects of walking and balance on a scale from 1 (“not at all”) to 5 (“extremely”). It is common to transform the questionnaire’s summed points (min. 12, max. 60) into a definite MSWS-12 score (min. 0, max. 100). A MSWS-12 score of 0 (= 12 summed points) means no impairment, a score of 100 (= 60 summed points) corresponds to highest possible gait impairment.

The MSWS-12 is a valid and widely used PROM to assess walking disability in pwMS and numerous studies confirmed its psychometric quality [14]. In several larger samples of pwMS (n > 100), correlations were seen between the MSWS-12 score and the EDSS, walking distance in 6 MW, gait parameters from quantitative gait analysis, the physical scale of the „Multiple Sclerosis Impact Scale“ (MSIS-29) and the patients’ daily physical activity assessed with accelerometry [2, 15–18]. In addition, benchmarks for the MSWS-12 score were identified regarding everyday functioning of pwMS. A score of 25 indicated beginning impairment of housekeeping while a score of 75 predicted severe limitations in essential daily tasks [19]. The MSWS-12 was also shown to be sensitive to improvements of mobility after physical rehabilitation in pwMS [20] and a reduction of the score ≥ 8/100 points has been suggested as a clinically relevant improvement of walking ability in pwMS under Fampridine therapy in an distribution- and anchor-based analysis with Patient Global Impression of Change (PGIC) and EuroQoL 5-Dimension 5-Level (EQ-5D-5 L) questionnaires [21]. On account of that broad evidence, the MSWS-12 has been used as an outcome in interventional studies with pwMS, concerning e.g. Fampridine [22, 23] or transcranial brain stimulation [24].

The original MSWS-12 has been translated into several languages to allow usage in non-English speaking pwMS, such as Italian, Persian and Brazilian Portuguese [25–27]. Of note, numerous non-validated translations may exist and may have been applied in clinical trials.

To date no validated German version of the MSWS-12 exists, limiting its application in German speaking pwMS. Alternative usage (e.g., interview-based) holds a risk of translation failures or differences in interpretation and connotation of the items. This can lead to a poor validity of the score and limits its comparability to international data. Furthermore, when asking patients about their perception of health and disease, differences in culture and everyday living conditions must be considered (e.g., public transport infrastructure, housing etc.) and require cultural adaption along with translation.

The aim of this study was to develop a linguistically valid version of the MSWS-12 in German language (MSWS-12/D). Linguistically valid means that the translated questionnaire is adapted to the target population and can be expected to provide the same informative value as the original version. The translation was performed in a procedure that was modified from guidelines for the cross-cultural adaption of PROMs suggested by Beaton et al. [28]. The second step of the adaption process was to evaluate the psychometric properties of the novel MSWS-12/D in pwMS to confirm its validity and comparability to the original version. We examined floor and ceiling effects, internal consistency, item level response patterns and convergent construct validity of the MSWS-12/D score. Results support equivalence between translation and original version.

## Methods

### Translation and linguistic validation

The process of translation and linguistic validation of the MSWS-12 was based on guidelines for the cross-cultural adaption of PROMs [28] which we modified in some points (in detail: Step 3 – only one back-translation by English native professional translator, Step 5 – pre-test in 20 pwMS only).

The process consisted of the following steps:

Step 1: Team of experts: Initially, a multi-centric German team of experts was formed, consisting of physicians and nurses experienced with MS, psychologists, physiotherapists, and medical students.

Step 2: First meeting and prototype: Members of the expert team provided two independent German translations of the MSWS-12. A professional translator (German native speaker) provided a third German version. Based on these three versions, the expert team agreed on a first *prototype* for the MSWS-12/D.

Step 3: Back-translation of prototype: The *prototype* was then back-translated into English by another professional translator (English native speaker) without medical background nor knowledge of the original MSWS-12.

Step 4: Second meeting and preliminary consensus: Based on all prior translations the team of experts together with both translators agreed on a *preliminary consensus version* of the MSWS-12/D.

Step 5: Pre-test: To evaluate acceptance and comprehensibility, the *preliminary consensus version* was pre-tested in a sample of 20 pwMS in academic MS centers. After completing the scale, participants answered pre-prepared questions about their understanding of the questionnaire and interpretation of single items.

Step 6: Third meeting and finalization: The results of the pre-test were again discussed in the team of experts and the finale *MSWS-12/D* was agreed on.

### Psychometric validation

#### Patients

In order to evaluate the psychometric properties of the novel MSWS-12/D the questionnaire was implemented in several ongoing prospective studies in two academic MS centers (three single-center longitudinal observational cohorts: CIS and ViMS at Charité-Universitätsmedizin Berlin and Hamburg MS patient database (HAPIMS) at the University Medical Centre Hamburg-Eppendorf (UKE) [29]; one single-center study on effects of rehabilitation intervention: AMBOS study at the Institute of Neuroimmunology and Multiple Sclerosis (INIMS) [30] of the University Medical Centre Hamburg-Eppendorf (UKE)). The studies included adult pwMS (all forms, according to revised diagnosis criteria [31]) covering a wide spectrum of disease severity. Per protocol, study visits were performed in stable phase to exclude effects of acute relapse. Clinical data were acquired during inpatient visits and participants completed the MSWS-12/D on site. Participants declared their written consent. Only one datapoint per subject was included into analysis.

For our analysis, we applied additional selection criteria to these datasets including only persons diagnosed with MS (all forms, according to revised diagnosis criteria [31], Clinically Isolated Syndrome (CIS) excluded), age between 18 and 70 years, ability to walk at least few meters with or without assistive devices (EDSS ≤ 7.0) and full data available for confirmed diagnosis of MS, MSWS-12/D and EDSS. Exclusion criteria comprised the inability to read or understand and follow study rules and other conditions that might interfere with walking ability.

At the Institute of Neuroimmunology and Multiple Sclerosis (INIMS) of the University Medical Centre Hamburg-Eppendorf (UKE) the AMBOS study was approved by the Hamburg Chamber of Physicians’ ethics committee (Registration Number PV5408). At Charité Universitaetsmedizin Berlin, the observational studies were approved by Charité ethics committee (CIS: EA1/182/10 and VIMS: EA1/163/12).

By pooling of data from different studies we aimed at a sample size of ≥ 100 as recommended for the psychometric evaluation of health status questionnaires (n ≥ 100 for internal consistency and n ≥ 50 for floor and ceiling effects and construct validity) [32]. Further, this procedure was expected to achieve a wider spectrum of disease severity and increase generalizability of results. The number of pwMS included in the pre-test sample was chosen according to common practice in qualitative research, where saturation of information is expected at numbers ≤ 20 [33].

#### Assessments

##### MSWS-12/D

Participants were asked to complete the novel translated MSWS-12/D on site, either on paper or on a tablet. Participants had the option to address the study team for questions on procedures, but no questions about the content of the MSWS-12/D were answered at this stage.

To transform the summed points of the MSWS-12/D (min. 12, max. 60) into a definite MSWS-12/D score (min. 0, max. 100), we used the following formula:

##### ((MSWS-12/D summed points − 12) / 48) x 100 = MSWS-12/D score

This transformation is proposed and commonly used for the original version [2]. A MSWS-12/D score of 0 (= 12 summed points) means no impairment, a score of 100 (= 60 summed points) corresponds to highest possible gait impairment.

##### EDSS

The severity of the disease was scored by a trained physician using the Expanded Disability Symptom Scale (EDSS). The EDSS is a widely used tool to assess disability in pwMS, rating seven different functional systems (pyramidal, cerebellar, brainstem, cerebral, sensory, bowl and bladder, visual) plus ambulation. An EDSS of 0.0 means no disability in any functional system, a score of 1.5 denotes the threshold of minimal signs without disability and 10.0 denotes death caused by MS. Considering walking ability, important benchmarks are EDSS 4.0 (beginning impairment in ambulation) and 7.5 (patient unable to walk more than few steps, wheelchair-bound). Persons with an EDSS 0.0–7.0 should be able to walk few meters with or without assistive device [4].

##### T25FW

The Timed 25 Foot Walk (T25FW) was assessed as part of the Multiple Sclerosis Functional Composite (MSFC) [34, 35]. The T25FW is a standardized stopwatch test of maximum walking speed over a 25 feet (7.62 m, m) distance. It is considered a valid tool to assess walking disability [14]. Walking speed (in meters per second, m/s) was derived from the time (in seconds, s) participants needed to walk the 7.62 m distance, averaged over two trials.

#### Statistical analysis

We evaluated the following psychometric properties of the novel MSWS-12/D:

*Floor and ceiling effects* describe the proportion of participants that obtain the highest or lowest possible score or results in a test. Optimally, the percentage of these results is less than 15% [32, 36].

*Internal consistency* defines the ability of a questionnaire and its single items to reliably measure a certain concept, here walking ability. It is usually specified by Cronbach’s alpha (α) coefficient which measures the homogeneity of this construct. A coefficient between 0.7 and 0.95 is rated positive [32]. A low Cronbach’s alpha (α) indicates poor correlation between the items concerning the measurement of this construct, whereas a very high coefficient indicates redundance in items [32].

*Convergent construct validity* is tested by correlations between the test results and other parameters that are considered to measure the same or a reasonably related construct [32]. In line with previous validation studies, we chose to correlate the MSWS-12/D score to a disability score known to rely on gait function (EDSS) [2, 17, 37] as well as maximum walking speed (T25FW) [18, 37]. Correlation of the MSWS-12/D score with the clinical severity of disease (EDSS) was examined using bivariate Spearman’s rho (ρ). For correlation of MSWS-12/D scores with maximum walking speed derived from T25FW we applied bivariate Pearson coefficient (r). We expected positive correlations between MSWS-12/D score and EDSS and negative correlations between the score and maximum walking speed.

For all data processing and analysis, we utilized IBM® SPSS Statistics 27.

## Results

### Translation and linguistic validation

The translation process (steps 1–3) revealed no major discrepancies considering the overall translation of the questionnaire. There were incongruencies between members of the expert team considering the translation of item 4 (“standing when doing things”) and item 11 (“smoothness of walking”). In the second meeting (step 4), the team of experts agreed upon a *preliminary consensus version* of the MSWS-12/D and decided to pay special attention to the comprehensibility of items 4 and 11 in the following pre-test process.

The pre-test of the *preliminary consensus version* was performed among 20 pwMS (step 5). After filling in the questionnaire, participants received a pre-prepared interview asking about:

1) general understanding of the MSWS-12/D (“Do you understand the wording of the questionnaire?” and “Are there items that you find difficult to understand?”),

2) interpretation of item 4 (“What situations come into your mind when you read ‘standing when doing things’”?) and.

3) interpretation of item 11 (“What comes into your mind when you read ‘smoothness of walking’”?).

For point 1), none of the 20 pwMS declared problems in understanding the questionnaire in general or single items. Point 2) about item 4 mainly resulted in answers such as “doing the dishes”, “preparing meals / cooking” or “folding laundry”. Point 3) about item 11 was interpreted as “without spasticity”, “regular / consistent / steady” or “without unevenness”.

**Fig. 1:**
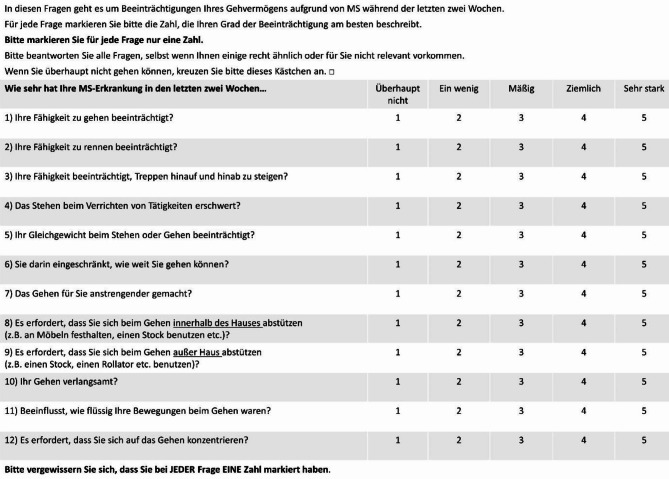
MSWS-12/D as a linguistically validated translation. *MSWS-12/D*: German version of the Multiple Sclerosis Walking Scale 12

After discussing the results of the pre-test in a third meeting (step 6), the expert team agreed upon the *preliminary consensus version* to be the final *MSWS-12/D* (Fig. 1).

### Psychometric validation

#### Patients

We obtained data from 124 pwMS (Table 1). Most of the persons had rather mild clinical symptoms (81% with EDSS < 4.0), and median EDSS was 2.0. Mean MSWS-12/D score was 24, with a median of 8.

**Table 1:** Group characteristics and MSWS-12/D scores of study sample

	n = 124
**age (years)**	
mean (SD); min. - max.	42 (11); 19–66
missing n (%)	1 (0.8)
**female** n (%)	77 (62.1)
**male** n (%)	44 (35.5)
missing n (%)	3 (2.4)
**disease duration (months)**	
mean (SD); min. - max.	135 (101); 0–468
missing n (%)	1 (0.8)
**MS disease course**	
RRMS n (%)	97 (78.2)
SPMS n (%)	17 (13.7)
PPMS n (%)	9 (7.3)
missing n (%)	1 (0.8)
**symptom severity**	**EDSS**
median	2
min.	0 (n = 8; 6.5%)
max.	7.0 (n = 1; 0.8%)
mild	< 4.0 (n = 100; 80.6%)
moderate - severe	≥ 4.0 (n = 24; 19.4%)
**max. walking speed (m/s)**	
mean (SD), min. - max.	1.59 (0.39); 0.34–2.9
missing n (%)	9 (7.3)
**MSWS-12/D score**	
median	8
mean (SD)	24 (31)
min.	0 (n = 45; 36.3%)
max.	100 (n = 1; 0.8%)

Group characteristics and MSWS-12/D scores of study sample. *EDSS*: Expanded Disability Status Scale, *max.*: maximal, *min.*: minimal, *m/s*: meters per second, *MSWS-12/D*: German version of the Multiple Sclerosis Walking Scale 12, *PPMS*: Primary Progressive Multiple Sclerosis, *RRMS*: Relapsing-Remitting Multiple Sclerosis, *SD*: Standard Deviation, *SPMS*: Secondary Progressive Multiple Sclerosis

#### Floor and ceiling effects

Distribution of MSWS-12/D total scores is shown in Table 2. For reasons of readability and clarity, we chose to visualize obtained MSWS-12/D scores in intervals of 10.

Specifically, floor effects were observed in 45 pwMS (36%, EDSS 0–3.5) who scored 0 in the MSWS-12/D. Only one pwMS (1%, EDSS 7.0) obtained a score of 100.

**Table 2:** Distribution of MSWS-12/D scores in study sample

MSWS-12/D score (intervals of 10)	n (%)
0	45 (36.3)
1–10	23 (18.5)
11–20	13 (10.5)
21–30	7 (5.6)
31–40	3 (2.4)
41–50	6 (4.8)
51–60	6 (4.8)
61–70	3 (2.4)
71–80	5 (4.0)
81–90	8 (6.5)
91–99	4 (3.2)
100	1 (0.8)

*MSWS-12/D*: German version of the Multiple Sclerosis Walking Scale 12

#### Internal consistency

For internal consistency we calculated a Cronbach’s alpha (α) of 0.98 for the MSWS-12/D.

**Fig. 2:**
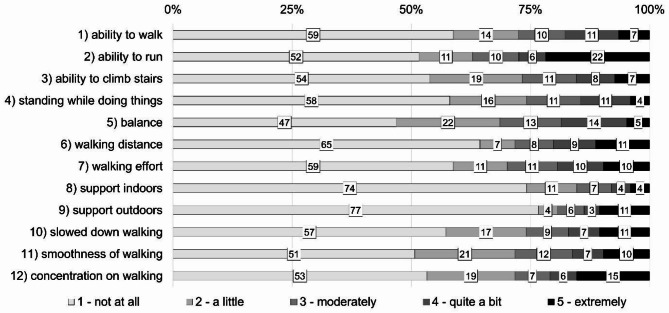
Distribution of responses (%) for single items of the MSWS-12/D. *MSWS-12/D*: German version of the Multiple Sclerosis Walking Scale 12

Response patterns for the single items are shown in Fig. 2. We only conducted descriptive analysis. More than half of the pwMS in our study sample declared problems with balance (item 5), and more than 45% stated limitations in item 2 (ability to run), item 3 (ability to climb stairs), item 11 (smoothness of walking) and item 12 (concentration on walking). More than one fifth of the pwMS declared to be “extremely” limited in their ability to run (item 2) and 15% stated the highest possible impairment in item 12 (concentration on walking). Necessity to use support (item 8 and 9) obtained lowest scoring.

#### Convergent construct validity

**Fig. 3:**
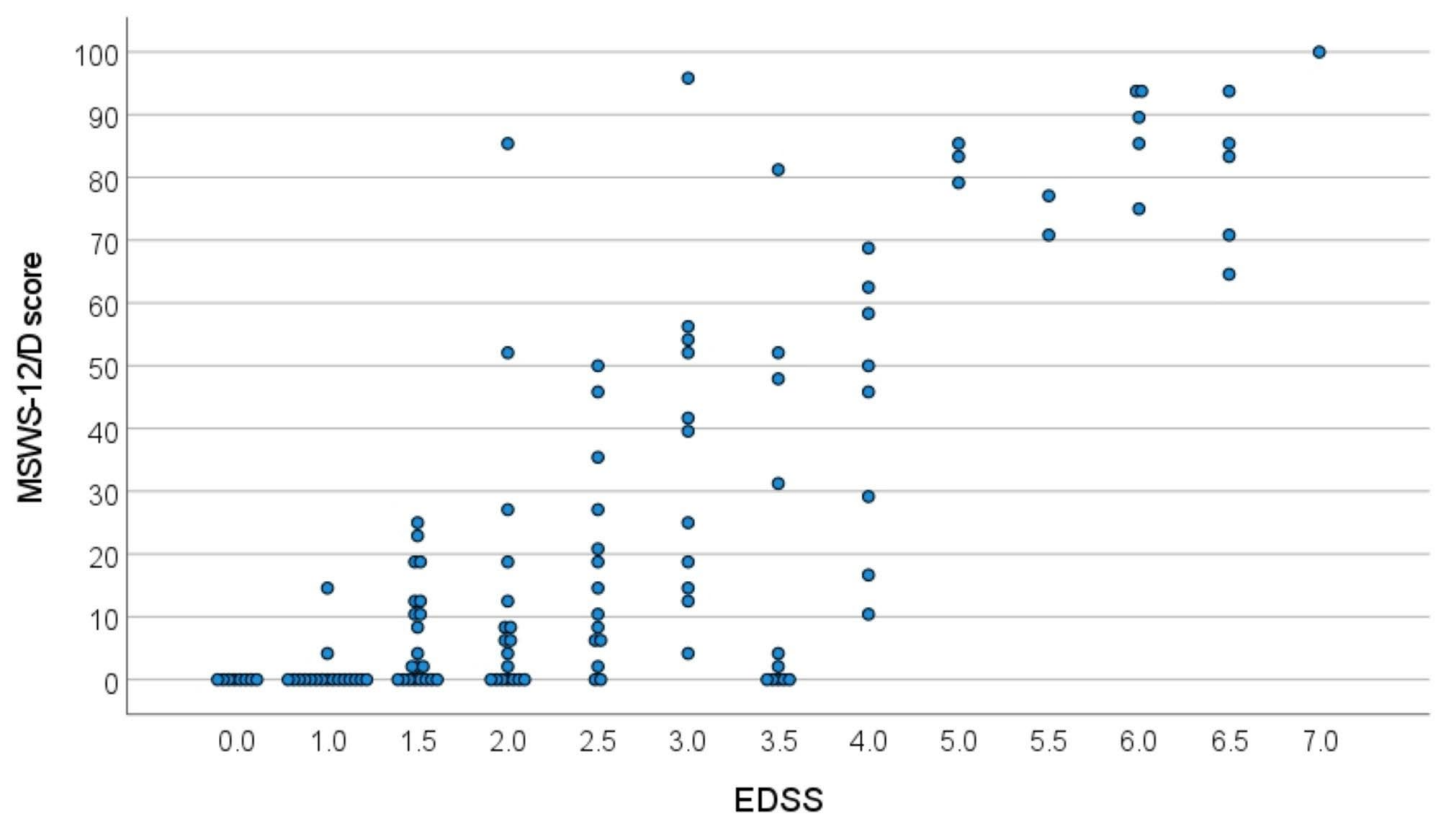
Scatter plot for correlation analysis between MSWS-12/D score and EDSS (n = 124). Identical data pairs are depicted adjacently. *EDSS*: Expanded Disability Status Scale, *MSWS-12/D*: German version of the Multiple Sclerosis Walking Scale

**Fig. 4:**
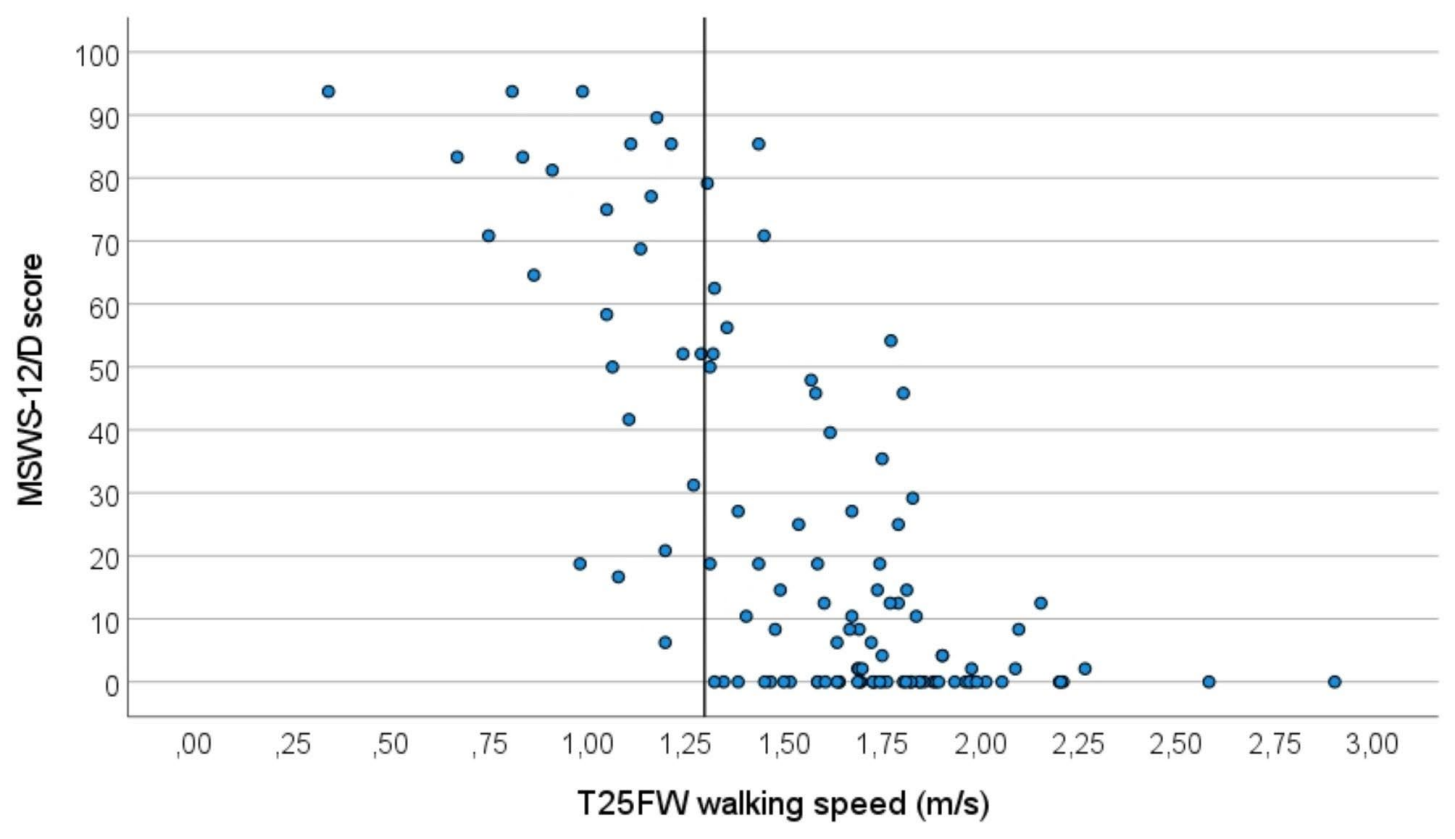
Correlation between MSWS-12/D score and maximum walking speed (in m/s) (n = 115). Reference line at 1.3 m/s. *m/s*: meters per second, *MSWS-12/D*: German version of the Multiple Sclerosis Walking Scale 12, *T25FW*: Timed 25 Foot Walk

We found relevant correlations between the MSWS-12/D score and the EDSS step as an overall rating of symptom severity (ρ = 0.731, p < 0.01) (Fig. 3) and the maximum walking speed (m/s) derived from T25FW (r =-0.716, p < 0.01) (Fig. 4).

We noticed two individual cases with low MSWS-12/D scores (< 20) despite an EDSS ≥ 4.0 and thus presumably at least moderate gait impairment (see Fig. 3). Inspection of source data in these two cases revealed, that one (38 years, RRMS, EDSS 4.0, MSWS-12/D score of 10) featured high EDSS sub scores in the visual functional system (grade 6), causing a high total EDSS score. The other pwMS (48 years, PPMS, EDSS 4.0, MSWS-12/D score 17) had scores of 2 assigned in all functional system of the EDSS except for sensory function (score of 1) and ambulation (fully ambulatory), and stated “moderate” limitations (3 points on MSWS-12/D scale) in items 1 (ability to walk), 10 (slowed down walking), 11 (smoothness of walking) and 12 (concentration on walking) while no limitations in the other aspects were stated. Vice versa, two pwMS in our sample with only signs according to EDSS = 1.0 scored > 0 in MSWS-12/D (see Fig. 3). According to medical records, both declared fatigue as a relevant symptom.

Data for maximum gait speed derived from T25FW was missing from 9 pwMS (7.3%). Median EDSS from this subgroup was 2.0, with 6 pwMS with EDSS range 0–3.5 and three pwMS with an EDSS between 4.0 and 7.0. Mean MSWS-12/D score was 37 with a range between 0 (n = 1; EDSS 1.5) and 100 (n = 1; EDSS 7.0), median score was 10. Two of these pwMS had the highest MSWS-12/D scores in our overall study sample (MSWS-12/D score 100 (EDSS 7.0) and 96 (EDSS 4.0)).

## Discussion

In this study, we provide for the first time a German version of the MSWS-12 translated in a multi-professional team of experts based on guidelines for intercultural adaption of PROMs [28]. Results from a multi-center validation process in 124 pwMS indicated that the psychometric qualities of the MSWS-12/D are comparable to those published for the original version. Relation to common ratings of disability would support its use as a screening tool as well as an instrument to monitor a relevant aspect of the disease course in MS.

Importantly, we transformed the questionnaire’s summed points (12–60) to a MSWS-12 total score (0 to 100) as proposed by Hobart et al. for their original version [19, 38]. However, not all publications using the MSWS-12 specify if and how they transformed the points of the questionnaire which impedes interpretation against the literature.

With respect to psychometric properties, results fit well to those reported for the original version or other published translations. For internal consistency we calculated a Cronbach‘s alpha (α) of 0.98 (original MSWS-12 α = 0.97 [2, 17]). In our sample of pwMS with an imbalance towards mildly affected (EDSS median 2.0) we observed relevant floor effects. This may be interpreted as MSWS-12/D being less sensitive in pwMS with mild symptoms, as suggested previously [39]. In contrast, other authors described even better sensitivity in pwMS with mild disease severity (EDSS 1.0–4.0) [40] and our results would support this. Concluding from inspection of the data plots (Fig. 3), pwMS with EDSS below 1.5, i.e., below the threshold of symptoms, consistently rated zero on MSWS-12/D except for two subjects. This implies that MSWS-12/D ratings of > 0 accurately indicate disease-related changes in walking to some extent. Nevertheless, in our study sample with only mild disability in the majority (81% with EDSS < 4.0) still about half of the pwMS stated problems with balance (item 5, 53%), smoothness of walking (item 11, 49%), ability to run (item 2, 48%) and need to concentrate on walking (item 12, 47%). In this sense, higher floor effects in samples of mild MS can be considered an accurate description of lack of disability and not necessarily a psychometric weakness. Our findings rather suggest potential utility of MSWS-12/D to screen for incipient disability in MS.

The MSWS-12/D covers aspects clearly beyond the performance level, such as concentration while walking, perception of effort while walking and smoothness. Subjects may endorse these items even though gross walking performance, walking speed or walking distance are (still) unimpaired.

If, however, results support specificity for disease-related limitations in walking ability, the MSWS-12/D may also be used to derive benchmarks for other related constructs. In this sense, a maximum walking speed of < 1.3 m/s can be considered to indicate a benchmark for patient-perceived limitations of walking ability, as none of the participants rated 0 on MSWS-12/D in this segment (Fig. 4). MSWS-12/D may thus have role for the validation of upcoming quantitative measures related to walking ability, such as instrumented analysis of gait and balance, activity trackers or mobility profiles. To our knowledge, there are no investigations that described the utility of MSWS-12 in these contexts, although some reported on relations of higher MSWS-12 scores with decreased daily physical activity assessed with accelerometry [15–17] and severe limitations in essential daily tasks [19]. Kalron et al. focused on the correlations between single items of the MSWS-12 and several clinical mobility tests and found that results for item 8 and item 9 (use of support indoors / outdoors) had the most informative value against clinical measures of walking and mobility [41]. A recent investigation suggested relevance of MSWS-12 for the detection of fall risk in pwMS [42]. The majority of fallers scored 40 or higher on MSWS-12 (mean 43 vs. 27 in non-fallers).

As described for the original version, our analysis showed relevant correlations between patients’ reported limitation in the MSWS-12/D and rater-based assessment of neurological impairment in the EDSS (ρ > 0.7). These findings resemble the results published for the original MSWS-12 (ρ = 0.65 [2] and ρ = 0.78 [17]).

For maximum walking speed, we observed a relevant correlation between MSWS-12/D score and maximum walking speed in T25FW (r=-0.72). Previous reports were less consistent in this respect and described correlations between the original MSWS-12 and T25FW (walking speed* or time**) between − 0.2* and 0.78** [14, 37, 43]. One possible cause is the inconsistent reporting of T25FW as speed or time and a possibly non-linear decline of this measure over the disease course. Further, the underlying T25FW construct of maximum walking capacity assessed at a single time point is expectedly less tightly related to patients’ perceptions of their general walking ability. This also explains limited ecological validity previously described for T25FW [7]. These aspects underline the immense relevance of PROMs as assessment tools for symptom severity in neurological diseases that should be applied complementary to performance-based clinical or instrumental ratings.

In our analysis we noticed an incongruence of two data pairs with low MSWS-12/D score < 20 but relevant disability according to EDSS. In one, the discrepancy might be explained by a high EDSS sub score for the visual functional system causing a total EDSS 4.0 while in the other, total EDSS was 4.0 due to the number of functional system ratings and ambulation was unrestricted. In contrast, two subjects with presumably signs only (EDSS = 1.0) scored > 0 in the MSWS-12/D but reported relevant fatigue as possible explanation. However, we cannot exclude the possibility of faults or errors occurring in filling in the questionnaire, as there was no immediate control of plausibility on site. Data for maximum walking speed derived from T25FW was missing from 9 pwMS, of which two had the highest MSWS-12/D scores in our overall study sample (MSWS-12/D score 100 (EDSS 7.0) and 96 (EDSS 4.0)).

A limitation to our translation process is the fact that we used a modified version of the guidelines for cross-cultural adaption of PROMs suggested by Beaton et al. [28]. In detail, in step 3 back-translation of the prototype was performed solely by one English native professional translator instead of at least two. This may have affected the process of detecting translation errors in the prototype as discussion between English native speakers was impossible. Other than recommended by Beaton et al., in step 5 we performed the pre-test in 20 pwMS (30–40 are suggested [28]). However, this number is in line with recommendations for qualitative research [33] and our pre-test with 20 pwMS showed a good acceptance and revealed no difficulties in comprehensibility of the questionnaire or single items. Results for the controversial items 4 and 11 were favorable.

We solely used data pairs from pwMS who filled in the MSWS-12/D completely. We hence cannot report about missing data, but very low rates have been reported by others (9 out of 293 total MSWS-12 records [44],). Further, this sample selection criterion holds the risk of convenience sampling bias. Subjects with conditions that interfere with filling in the questionnaire (e.g., limitations in hand usage or psychological conditions such as fatigue) could have refrained from participating. However, refusal to complete the questionnaire or other PROM as part of the protocols was not observed among participants of the contributing studies with rather mild impairment in the majority. To extend the validity of the novel MSWS-12/D, further studies are needed focusing on pwMS with more severe levels of disability, as comorbidities such as fatigue are more likely to occur in this group [3]. In the present study we solely examined certain psychometric properties (floor and ceiling effects, internal consistency, construct validity) of the MSWS-12/D. We suggest additional evaluation of the score, regarding qualities such as test-retest reliability or longitudinal measurement invariance.

As a general limitation to our work, psychometric evaluation was confined to methods of Classical Test Theory (CTT). Recent studies applied and recommended using Item Response Theory (IRT) [44–46]. However, their revised IRT-based scoring system showed associations with T25FW speed highly similar to the usual MSWS-12 score (r=-0.71 and 0.70, respectively), but some minimization of error might be achieved in the MSWS-12 ranges of < 20 and > 80. IRT seeks to detect differences in the function of single items for the overall test. Problems were found regarding the functioning of items 2, 8 and 9 that were rarely rated as “most likely” [44–46] and age was described as a measurement bias for item 2 [44]. Consistent with those findings, we also saw differing response patterns for items 2, 8 and 9, but this was not analyzed statistically. Interestingly, the IRT approach has also been suggested to check for plausibility of individual data using IRT-based likelihood of individual item responses [44].

From March 2018 to July 2018 the novel MSWS-12/D was part of a scientific online survey for pwMS with a history of falls / self-perceived risk of falling, which was conducted by the UKE Hamburg (ethical approval by Hamburg Chamber of Physicians, reference number: PV 5609) and was accessible at the website of the patient organization German Society of Multiple Sclerosis / Deutsche Multiple Sklerose Gesellschaft (DMSG) ( [47], Kesgin et al., 2021, unpublished work). Due to the fact that MS diagnoses as well as disability ratings were not clinically confirmed in this survey, we chose not to include data derived from this survey in the validation process of this work. Yet we briefly want to summarize findings in this more severely disabled cohort. Until July 2018, 310 pwMS with a self-perceived risk of falling participated in the survey. Median PDDS was 4 with 80% of the participants with an PDDS ≥ 3, i.e., manifest gait impairment [12, 13]. In this online survey group, mean MSWS-12/D score was 61 and expectedly higher than in our validation sample with mean lower disability. Bivariate Spearman’s correlation between the MSWS-12/D score and the PDDS of ρ = 0.832 (p < 0.01) was similar to results of the original MSWS-12 (ρ = 0.8 [12]).

“Walking ability” is a complex construct, consisting of patients’ mere body functions and performance but also real-life capacity and habits of mobility. The MSWS-12 was developed as a simple measure of walking ability in pwMS, asking about different aspects of standing and walking. Studies confirmed that the score measures the single-factor “walking ability” adequately [17]. Our results support MSWS-12 as well applicable and informative over a wide range of disability in pwMS and thus its utility to track the disease course with the charm of remote application. Data also support its potential as an easily applicable screen for incipient disease related limitations. As an extension to the MSWS-12, Holland and colleagues developed the “Walk-12” as a generic version of the questionnaire and confirmed its validity in patients with other neurological diseases (stroke, spinal cord injury) [48]. Subsequently, the Walk-12 has been used in different neurologic disorders, such as Parkinson’s disease [49], poliomyelitis [50] or stroke [51].

To improve interpretation of individual MSWS-12/D scores and to investigate their informative value concerning specific features of disease, we suggest examining the score’s relation to aspects of everyday function and quality of life outcomes as well as specific neurological symptoms, motor and non-motor features or patterns of structural lesions in pwMS.

## Conclusion

In this study we developed a German version of the MSWS-12 (MSWS-12/D). The MSWS-12/D showed good psychometric quality and can be applied in German speaking persons with Multiple Sclerosis to assess walking ability from the patient’s perspective.

## Data Availability

The datasets used and analyzed during the current study are available on reasonable request.

## Abbreviations

6MW: 6 min Walk
CIS: Clinically Isolated Syndrome
CTT: Classical Test Theory
DMSG: German Society of Multiple Sclerosis / Deutsche Multiple Sklerose Gesellschaft
DS: Disease Steps
EDSS: Expanded Disability Status Scale
EMA: European Medicines Agency
EQ-5D-5L: EuroQoL 5-Dimension 5-Level
FDA: U.S. Food and Drug Administration
HAPIMS: Hamburg MS patient database
INIMS: Institute of Neuroimmunology and Multiple Sclerosis, Center for Molecular Neurobiology, University Medical Center Hamburg-Eppendorf
IRT: Item Response Theory
m/s: meters per second
max.: maximal
min.: minimal
MS: Multiple Sclerosis
MSFC: Multiple Sclerosis Functional Composite
MSIS-29: Multiple Sclerosis Impact Scale 29
MSWS-12/D: German version of the Multiple Sclerosis Walking Scale 12
MSWS-12: Multiple Sclerosis Walking Scale 12
NCRC: Neuroscience Clinical Research Center, Charité-Universitätsmedizin Berlin
PDDS: Patient Determined Disease Steps
PGIC: Patient Global Impression of Change
PPMS: Primary Progressive Multiple Sclerosis
PROM(s): Patient Reported Outcome Measure(s)
pwMS: Persons with Multiple Sclerosis
RRMS: Relapsing-Remitting Multiple Sclerosis
SD: Standard Deviation
SPMS: Secondary Progressive Multiple Sclerosis
T25FW: Timed 25 Foot Walk
UKE: University Medical Centre Hamburg-Eppendorf
